# The Approach of Age

**Published:** 1885-08

**Authors:** 


### The Approach of Age.

The approach of age shows itself about the eyes. Lines come, faintly at first, then deeper, until the incipient crows' feet are indicated, developed and revealed. The woman, who, looking in her glass, sees these fatal lines, diverging from the outer corner of her eyes, knows that she has reached an era in her life. She recognises it with a sigh if she be a vain, a lovely or a worldly woman ; with a smile, perhaps, if she has children in whom she can live her own youth over again. But it can never be a gay smile ; none of us, men or women, like to feel youth—that precious possession—slipping away from us. But we should never be seen on the lookout for crows' feet or gray hairs. Looking for them is sure to bring them, for thinking about them brings them. Tears form a part of the language of the eye, which is eloquent enough when sparingly used, and which should be sparingly used for other reasons than that of adding to their mute eloquence. Tears are a disfiguring expression of emotion, and those who get in the habit of weeping over every small vexation do much to acquire a careworn, miserable expression, and are sure to look old before their time. Excessive weeping has been known not only to injure but acturlly to destroy the sight. Few women look pretty or even interesting in tears, though it has long been a pleasant fiction in poetry and romance to suppose that they do. Many women, some men, most children, make most disfiguring and distorting grimaces while crying ; and the lady who thinks she can work upon a man's feelings by a liberal display of tears, should carefully study a becoming mode of producing them before her looking-glass. Grimaces soften no heart, and tears, accompanied by the usual distortion, have a hardening effect if not a visible one.

In a prettily written work, now probably out of print, purporting to be the story of the life of one of Milton's wives, the author makes the poet say of his wife's eyes, after crying, that they resembled "the sun's clear shining after the rain," a very pretty natural object indeed, but during the rain itself the observer is not inclined to be complimentary.—*Whitehall Rev.*

